# 

**Published:** 1880-06-20

**Authors:** 


### TABLE OF CONTENTS.

ORIGINAL AND SELECTED ARTICLES.

Lepra, by Jas. H. Low, M. D., New York City, 193. Yellow Fever, a more Indigenous than Exotic Disease and not belonging to the Zymotic in the shape of Contagion, by L. V. Weathers, M. D., of Ark., 195. On Some Points in the Therapy of Aconite, by J. W. Hickman, M.D., Delta, Pa., 197. Cold Wet Sheet Pack in Scarlatina, by Dudley M. Culver, M. D., of Whitesville, Ind., 200. Chicken Cholera—Pasture's New Experiments, 201. Sulphide of Calcium in the Treatment of Supperating Buboes, by Fessenden N. Otis, M. D., 203. If Veratrum Viride is a sure Antidote for Opium Poisoning in the Acute Form, may it not be in Chronic, Opium Inebriety?—an Affirmative Opinion, by J. S. Haldeman, M. D., of Ohio, 205. Treatment of Acute Rheumatism, by J. Alexander Larue, M. D., of W. Virginia, 207. Recto-Vaginal Fistula Cured without Operation, by Ira Brown, M. D., Wells River, Vt., 208.

ABSTRACTS AND GLEANINGS.

The Active and Passive Inhalation of the Nascent Chloride of Ammonium in Acute Affections of the Respiratory Tract, 209. Advances in Materia Medica, 212. Treatment of Diseases of the Tonsils, 215. A case of Typhoid Fever trated with Carbolic Acid Internally, 216. The Cure of Cancer, 217. Purgatives—For what Objects shall they be Administered in Treating Cases of Diarrhea and Dysentery; When to Perform Ovariotomy, 218. The Dissemination of Typhoid Fever, 219. The Tongue; First Death from Bromide of Ethyl, 220. Treatment of Syphilitic Ulcerations by Pyrogallic Acid; Influence of Singing upon Health, 222.

SCIENTIFIC ITEMS.

The Sun's Rays as a Means of Research; Telephonic Transmission through the Human Body; Telephone in Scotland; Cold as a Motor, 223. Sanitas; More Scotch Diamonds; A Transparent Fish; Engineering Inventions, 224.

PRACTICAL NOTES AND FORMULÆ.

Cinchonia Alkaloid; Worms in the Nasal Cavities Expelled by Means of Chloroform, 225. Veratrum in Dysentery; Vermifuge; Tartrate of Morphia; For Dysentery; An Emetic for Infants, 226. Inflammation of the Middle Ear; Fresh Cold in the Head; Oleate of Lead in Eczema; Reducing a Paraphimosis; Richardson's Stypic Colloid, 227.

EDITORIAL AND MISCELLANEOUS.

Editorial Notices; Medical Editors' Association, 228. Annual Meeting of the American Medical Association, 229. Book Notices, 231. Receipted; Special Notices, 232.

